# Comparison of the PIVOT App and Pivot Shift Test for Assessing Knee Laxity after ACL Injury: Validity and Impact of Thigh Muscle Coactivity

**DOI:** 10.1177/23259671261469739

**Published:** 2026-08-12

**Authors:** Madison Landry, Matthew J. Jordan, Denise Chan, Jean-Michel Galarneau, Kati Pasanen, Volker Musahl, Nicholas Mohtadi

**Affiliations:** † University of Calgary Sport Medicine Centre, Calgary, Alberta, Canada; ‡ Faculty of Kinesiology, University of Calgary, Calgary, Alberta, Canada; § McCaig Institute for Bone and Joint Health, University of Calgary, Calgary, Alberta, Canada; ‖ Department of Surgery Cumming School of Medicine, University of Calgary, Calgary, Alberta, Canada; ¶ Integrative Neuromuscular Sport Performance Laboratory, University of Calgary, Calgary, Alberta, Canada; # Sport Injury Prevention Research Centre, University of Calgary, Calgary, Alberta, Canada; ** University of Pittsburgh Medical Center, Pittsburgh, Pennsylvania, USA; Investigation performed at the University of Calgary Sport Medicine Centre, Calgary, Canada

**Keywords:** anterior cruciate ligament, electromyography, knee injury, pivot shift test, thigh muscle coactivity

## Abstract

**Background:**

The pivot shift is an important and widely used orthopaedic test for assessing anterior cruciate ligament (ACL)-deficient (ACL-D) knees. A continuous measure of the pivot shift test may allow for a more objective assessment of ACL laxity.

**Purpose:**

To assess the efficacy of a novel iPad application (PIVOT App) that uses video image analysis to evaluate ACL deficiency. The main objectives were to (1) assess the PIVOT App's ability to distinguish between categorically assigned pivot shift test grades; (2) assess the PIVOT App's ability to differentiate between normal and abnormal knees, and (3) quantify the effects of thigh muscle coactivity on pivot shift grades using electromyography (EMG) analysis.

**Study Design:**

Cohort Study (Diagnosis); Level of evidence, 3.

**Methods:**

ACL-deficient (ACL-D) participants recruited via convenience sampling underwent the pivot shift test on their injured and contralateral knees. Some patients were evaluated in the clinic (n = 33), and others were evaluated under anesthesia (n = 20). Control participants (n = 7) with no history of knee injury also had the pivot shift test conducted on both knees while awake in the clinic. Tests were graded categorically by the examiner and simultaneously videotaped and analyzed by the PIVOT App, while EMG activity was recorded from the rectus femoris and semitendinosus muscles.

**Results:**

Pivot shift grades 1, 2, and 3 classified knees showed a consistent, incremental increase in tibial reduction. Grade 0 knees showed lower tibial reduction than grade 2 and 3 knees (*P* < .05). The PIVOT App's classification accuracy of normal versus abnormal knees was 73%. There were no effects of RF or ST EMG activity on the pivot shift test.

**Conclusion:**

The PIVOT App is a promising, noninvasive measuring device that can provide an objective, continuous assessment of the pivot shift test, addressing multiple limitations of the current reference standard.

Along with other clinical orthopedic tests used to assess knee laxity, such as the Lachman and anterior drawer tests, the pivot shift is an important diagnostic sign of an ACL deficient (ACL-D) knee.^15,31,34,35^ Unlike other tests, the pivot shift test measures not only tibial translation, but also lateral rotational instability.^22,29^ The pivot shift test provides clinicians with crucial information regarding knee joint laxity. However, there are certain limitations compared to other diagnostic examinations such as the subjective nature,^22,23,31^ inter-examiner variability including changes to standardization of testing maneuvers,^3,8^ and categorical grading schema of the test.^13,19,31^ A continuous measure of the pivot shift test may allow for a more objective assessment of ACL laxity.

The PIVOT App is an iPad application that uses video analysis to quantify tibial reduction during the pivot shift test as a continuous measure.^15^ The application is intended for use by medical professionals and solely for authorized research studies.^33^ Various devices have been researched in an attempt to produce an objective quantification of the pivot shift test, such as inertial measurement unit sensors, video image analysis, electromagnetic sensors, and computer navigation systems.^10,21,24-26,34^ However, research on the ability of these devices to assess ACL deficiency has predominantly been conducted in cadaveric studies^6,10,25^ or while patients are under general anesthesia.^21,23,26,34^ Additionally, there are multiple limitations to the use of these devices, including their applicability in clinical settings, invasiveness (eg, bony fixation), and cost.^15,17,25^ The PIVOT App offers an accessible, cost-effective, and portable way to assess ACL deficiency in a clinical environment.^15,23,25^

The pivot shift is more difficult to elicit when patients are awake due to voluntary protective guarding that arises from increased coactivity of the knee extensor and flexor (ie, thigh) muscles.^11,35^ Measuring muscle activity with electromyography (EMG) is a possible method to control for this added variability when conducting the pivot shift test on patients who are awake in a clinical environment. EMG analysis is a widely used tool to assess muscle activity in athletes with ACL-D.^1,4,7,9,20^ The combined use of surface EMG with clinical knee assessments as a means of quantifying patient guarding is also a novel contribution to the scientific literature with potential clinical applications.

The main objective of this study was to examine the validity and feasibility of the PIVOT App in a clinical sports medicine setting for assessing patients who were awake and under voluntary control of their skeletal muscles. A secondary objective was to examine the effects of patient guarding on the grade of the pivot shift when using the reference standard and the PIVOT App. This was done using EMG analysis to quantify coactivity of the thigh muscles, the rectus femoris (RF) and semitendinosus (ST), during the pivot shift test. The primary hypothesis was that the PIVOT App would be valid against the accepted reference standard in a clinical population. For the exploratory pilot using EMG analysis, it was hypothesized that thigh muscle coactivity, represented by increased EMG activity in the RF and ST muscles, would be associated with lower categorical grades and less tibial reduction reported by the PIVOT App.

## Methods

### Design

This was a diagnostic accuracy study to validate an iPad application (PIVOT App) for quantifying the pivot shift test in a clinical environment. Ethics approval was obtained.

### Participants

**Figure 1.**
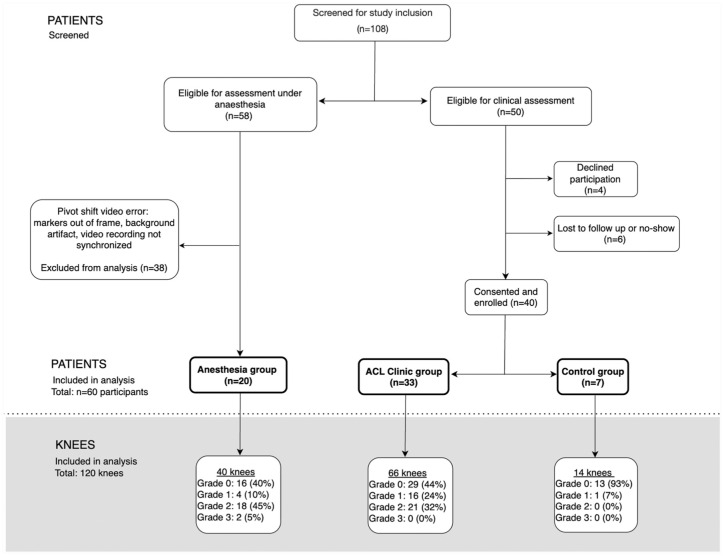
Flowchart of participants, dichotomized by the environment in which the pivot shift test was analyzed. A total of 108 participants were initially screened and assessed for study eligibility. Participants were not included in the analysis due to video-recording errors (markers out of frame), declined participation, or lost to follow-up. A total of 60 participants were eligible and included in the analysis: 20 were assessed in the operating room under anesthesia, 33 anterior cruciate ligament-injured patients, and 7 controls were assessed in the sports medicine clinic. In total, 120 knees were assessed with varying pivot shift grades.

A consecutive series of patients from an orthopaedic sports medicine practice with suspected unilateral ACL injury was approached to participate in the study (Figure 1). The inclusion criteria were as follows: age between 16 and 50 years, skeletally mature, with a suspected unilateral partial or complete ACL tear, as determined by an orthopaedic surgeon's clinical assessment, and either awaiting surgery or in the 3- to 12-month postoperative follow-up period. Patients were excluded if they had bilaterally injured knees, an inability to provide consent, inflammatory arthropathy or other systemic joint condition, a limitation of range of motion (ROM) precluding performance of a standardized knee examination (eg, a locked knee with loss of extension), or generalized pain.

A second group of patients was included from a concurrently conducted randomized trial comparing ACL reconstruction surgical techniques. These patients were all suspected of having ACL deficiency and met the same inclusion criteria. These patients had previously consented to the knee examination and PIVOT App measurements under anesthesia. A convenience sample of 20 patients was included, with complete data available from the operating room. A third group of control patients was approached from the same clinic. They were included in the same age group and had no history of any knee injury to either knee. Participants without knee injuries were included to evaluate a broader spectrum of patients with knee laxity, reflecting a realistic clinical scenario.

### Procedures

#### Participant Preparation

Participants were provided with a study overview and written informed consent forms. Researchers examined both knees, conducting baseline tests such as passive ROM, the Lachman test,^32^ valgus and varus assessment at 20° of knee flexion, knee circumference measurement, and Beighton score assessment.^27^ These values were assessed in the analysis as potential covariates.

#### Pivot Shift Test

The test procedure was identical for participants in all groups, including under anesthesia, ACL-injured patients in the clinic, and controls, to ensure consistency with the reference standard. To ensure optimal testing conditions for the PIVOT App, the examination room setup included positioning of 2 large softbox lights on either side of the patient examination table to replicate the operating room lighting. The pivot shift examiners included 2 experienced orthopaedic knee surgeons (including N.M.). They placed three 17-mm, circular, yellow reflective markers on the skin directly over the lateral epicondyle, fibular head, and Gerdy's tubercle on both knees.^15^ These markers were placed in identical positions in both the operating room and clinic settings. All yellow items were removed or covered from the field of view of the iPad to ensure PIVOT software would not mistake them for reflective markers, altering the analysis.

The examiner performed the pivot shift test using a modified flexion-rotation drawer method^3^ and applied a categorical grading from 0 to 3 based on the International Knee Documentation Committee (IKDC) criteria. ^8,11,32^ No accepted cutoffs of PIVOT App reduction values currently exist to classify knees into various grades. The pivot shift test was performed with optimal skill to minimize the effect of guarding in the clinical setting and to simulate, as closely as possible, the circumstances in the operating room under anesthesia. The examiners were blinded to the PIVOT App reduction measurements. The IKDC classification was used.^13^ A grade 0 (negative) or grade 1 (glide) pivot shift was considered “normal or nearly normal.”^13^ A knee with a grade 2 (clunk) or grade 3 (gross) was classified as “abnormal or severely abnormal,” respectively, and represented a confirmed ACL-D knee.^13^ Concurrently, the research assistant recorded a sagittal plane video of the pivot shift test with the iPad video camera. The research assistant ensured that the markers remained within the iPad's field of view throughout the test on both knees. The largest detected lateral translation value based on the relative movement of the reflective stickers to one another was recorded.^15^ Tibial reduction (mm), reduction time (s), elapsed time (s), and a scatter plot of femoral anteroposterior position from Gerdy's tubercle (mm) versus time (s) were obtained.

#### Surface EMG Recordings

The researcher shaved and cleaned the skin over the RF and ST muscles in accordance with the Surface Electromyography for the Non-Invasive Assessment of Muscles (SENIAM) procedures.^14,18^ Bipolar Ag/AgCl electrodes were secured to the skin (Norotrode, 2 cm interelectrode distance) and covered with tape to minimize movement artifacts and limit disruptions to video image analysis. Participants performed maximal voluntary contractions (MVCs) of isometric knee flexion and extension to normalize EMG.^18^ Participants received strong verbal encouragement and held the isometric MVC for 5 seconds. ^7^ The maximum accepted impedance was 7 kW. EMG data were collected using a personal computer (Noraxon MR3.20) with a sampling rate of 2000 Hz. A 100 ms centered root-mean-square filter was applied in the software, and peak EMG rms values were obtained from the RF and ST muscles.^18^ During testing, EMG data were synchronized with a continuous video recording of the knee (Noraxon, MR3.20, myoSync).

### Statistical Analysis

To assess tibial reduction between categorical grades, a multiple linear regression model was used with clustered-robust standard errors to account for the nonindependence in measurements from the same participant (ie, measurements from both legs of each person). A bivariate analysis explored the proportion of the variation in PIVOT App reduction explained by each independent variable, and surviving independent variables were included in the model if statistically significant (*P* < .05). Sex was added in all analyses as a potential confounder. Pairwise comparisons of marginal predicted means from the linear regression were used to further assess significant differences between adjacent categorical grades. Relationships between continuous variables and the PIVOT App reduction were tested with likelihood ratio tests and visually inspected *a priori* using a locally weighted scatterplot smoothing (LOWESS) curve.

To determine the PIVOT App's ability to correctly classify a knee as either normal or abnormal, measurements from the PIVOT App were entered into a multiple logistic regression as a continuous variable. As in the linear regression model, predictor variables were retained in the full model if their corresponding *P* values were statistically significant at *P* < .05. Sex was added in all analyses as a potential confounder. Predicted probabilities from the model were then used to produce classification statistics and the area under the receiver operating characteristic curve.

To determine the association between EMG activity and pivot shift test grade, a linear regression analysis was used to examine the effects of RF and ST muscle activity on the pivot shift test grade as the covariate of interest, as in the previous linear regression analysis. To determine the association between EMG activity and PIVOT App reduction output, a Spearman rank correlation analysis was first performed for both muscle groups, making no assumptions about the data. To graphically assess the data, LOWESS curves were used to evaluate potential trends before a linear regression was employed to measure the mean change in muscle activation for each unit increase in movement detected by the PIVOT App. All statistical analyses were performed using Stata 18 software (StataCorp LLC).^30^

## Results

A total of 60 participants were included in the analysis and underwent a pivot shift on both knees (Figure 1). Of the 120 knees assessed, 78 (65%) were normal, and 42 (35%) were abnormal. Further participant characteristics for each group are shown in Table 1.

**Table 1:** Participant Characteristics by 3 Groups and Environment Where the Pivot Shift Test Was Performed*^*a*^*

Participants	Anesthesia n = 20	ACL-Clinic n = 33	Controls n = 7
	Mean (SD)	Mean (SD)	Mean (SD)
Mean age, years	20 (2.88)	27 (8.84)	28 (6.64)
Beighton Score	3 (1.65)	3 (2.28)	3 (3.22)
	n (%)	n (%)	n (%)
Sex			
Male	8 (40)	14 (42)	3 (43)
Female	12 (60)	19 (58)	4 (57)

a The pivot shift test was analyzed in the clinic or the operating room. Controls were analyzed in the clinic. ACL, anterior cruciate ligament.

### Categorical Grades

**Figure 2.**
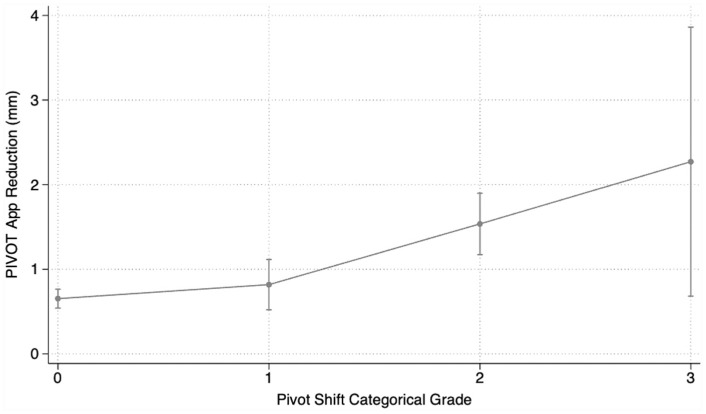
Adjusted tibial reduction from the PIVOT App (mm) by categorical pivot shift test grades with 95% CIs.

The mean reduction values from the PIVOT App showed a positive relationship with the categorically assigned grade. As the grade increased from 0 to 3, the mean reduction values increased respectively (0.62 ± 0.41 mm; 0.84 ± 0.65 mm; 1.56 ± 1.15 mm; and 2.39 ± 1.54 mm) (Figure 2). Results from the adjusted linear regression showed no difference in reduction between grade 1 and grade 0 knees (β = 0.165 mm [95% CI, −0.143 to 0.474]; *P* = .287). Both grade 2 (β = 0.884 mm [95% CI, 0.514 to 1.254]; *P* < .001) and grade 3 (β = 1.619 mm [95% CI, 0.033 to 3.206]; *P* = .046) knee reduction values were higher than those for grade 0, indicating increased translation. No statistically significant differences in sex were found (β = 0.122 mm [95% CI, −0.223 to 0.467]; *P* = .482). The full model explained 30% of the variance in the reduction in mm measured with the PIVOT App (*R*^2^ = 0.299).

The relationship between all combinations of grades was further assessed. Grade 1 was not statistically significantly different from grade 0 (β = 1.454 [95% CI, −0.170 to 3.078]; *P* = .078), grade 2 was statistically significantly different from grade 1 (β = 0.718 [95% CI, 0.276 to 1.160]; *P* = .002). Grade 3 knees showed larger tibial reduction than grade 2 knees; however, the difference was not statistically significant (β = 0.736 mm [95% CI, −0.906 to 2.377]; *P* = .373).

### Normal/Nearly Normal vs Abnormal/Severely Abnormal

**Figure 3.**
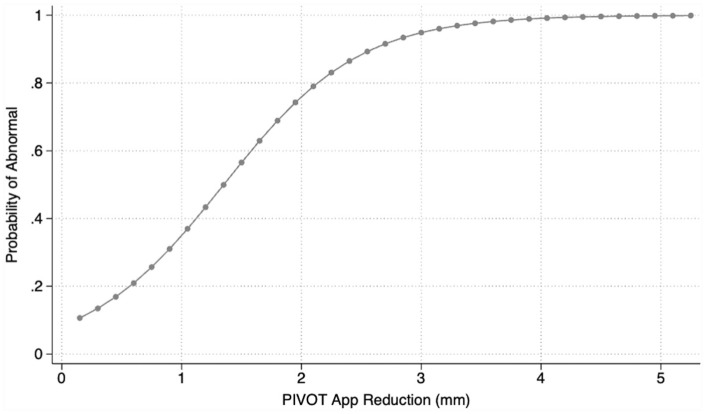
Probability of abnormal diagnosis against reduction values from the PIVOT App.

Reduction measured with the PIVOT App was strongly associated with knee abnormality (odds ratio [OR], 5.870 [95% CI, 2.200-15.695]; *P*≤ .001), indicating that for every 1-mm increase observed by the PIVOT App, the odds of the knee being classified as abnormal were 5.87 times higher. There was no association between sex and tibial reduction (OR_male_ = 1.040 [95% CI, 0.474-2.283]; *P* = .922). The relationship between millimeters of movement as measured by the PIVOT App and the probability of being classified as abnormal by manual categorical assessment is shown in Figure 3. The probability of having an abnormal knee was approximately 35% with just 1 mm of movement detected by the PIVOT App, while 3 mm of movement produced a probability of approximately 95%.

**Figure 4.**
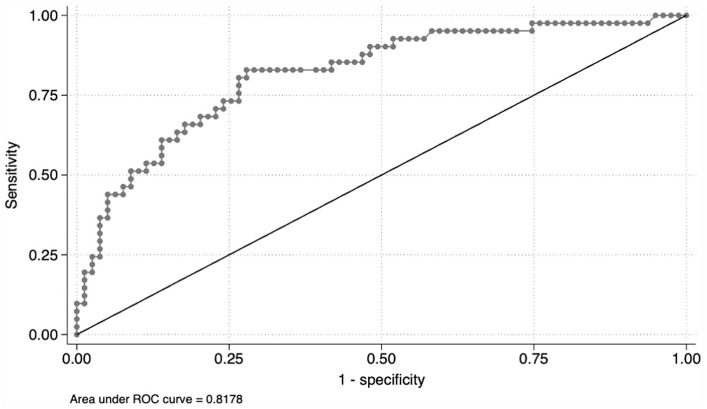
The AUC exhibits the outcome variable of knee abnormality based on the IKDC classification. AUC, area under the receiver operator characteristic curve; IKDC, International Knee Documentation Committee.

By fitting a multivariable logistic regression model for knee abnormality as the outcome variable with reduction (mm) and sex as predictor variables, the probability cut point was 29.2% (0.851mm), determined based on the intersection of highest sensitivity and specificity. Using this probability cut point, the PIVOT App successfully classified 73.33% of the cases as either normal or abnormal according to the previously described IKDC classification system.^15,17^ Sensitivity and specificity were similar, at 73.17% and 73.42%, respectively. The app exhibited a high negative predictive value at 84.06%; however, the positive predictive value was lower at 58.82%. As seen graphically in Figure 4, the area under the receiver operating characteristic curve for this predictive model was 81.78%.

### EMG Results

The mean MVC-normalized ST and RF muscle activity for participants analyzed in the clinical environment were 4.69% (4.38) and 3.37% (2.93), respectively (n = 38; including ACL-injured and control groups). The maximum value for MVC-normalized ST muscle activity, when a pivot shift could still be elicited by the examiner, was 16.03%, compared with 13.90% for the RF muscle. There was no correlation between EMG and knee laxity for RF (ρ = 0.0065; *P* = .954) or ST (ρ = −0.083; *P* = .461).

Results from the adjusted linear regressions indicated no associations between ST muscle activity and pivot shift grade (*R*^2^ = 0.003; *P* = .913). For the RF activity, a small, statistically significant relationship was found with categorical grade (*R*^2^ = 0.0625; *P* = .039). Notably, RF muscle activity was lower in grade 1 knees than in grade 0 knees (β = −1.672 mm [95% CI, −2.970 to −0.373]; *P* = .013). No other relationships were observed.

## Discussion

The PIVOT App showed a positive relationship with the categorical grades of the clinical pivot shift test and a linear increase in reduction from grades 1 to 3. Statistically significant differences were observed between grade 0 and grade 2, grade 0 and grade 3, and grade 1 and grade 2. Although there was no statistically significant difference between grades 0 and 1, or between grades 2 and 3, the effect size indicated a nearly perfect incremental increase from grade 1 to 2 and from grade 2 to 3. ST and RF muscle activity levels did not appear to impact the results obtained from the PIVOT App.

A total of 60 participants were included in this study (53 patients and 7 controls), representing 120 separate knee evaluations. It would be expected that there would be a minimum of 47 knees (20 contralateral knees from the operating room, 14 knees from the controls, and 33 knees from clinic patients) with a “0” grade or negative pivot shift, representing the uninjured contralateral knees. However, 21 knees were classified as having a grade 1 pivot glide. This raises the question of whether a pivot glide, whether determined by experienced examiners or measured by the PIVOT App, is a normal finding in some knees. The IKDC classification that summarizes knees into “normal or nearly normal” is supported by the study findings, but this did not reach statistical significance.

The pivot shift test is a widely used knee examination to assess anterolateral rotary instability in ACL-D knees.^16,22,35^ Although this commonly used test can provide health practitioners with a great deal of information, there are multiple limitations to the test. The PIVOT App addresses multiple issues with current manual testing methodologies, including potential intertester differences in standardization, subjective examiner perceptions of the pivot grade, and broad categorizations of injury severity.^13,16,22^ Using the video analysis software, the PIVOT App calculated the millimeters of tibial reduction during the pivot shift test maneuver. The PIVOT App produces a numerical, continuous value, as opposed to the reference standard, which is largely based on examiner perception and on classifying injury severity into 4 categorical injury grades.^15,19^ Similar to the KT-1000 device for the Lachman test,^8,28^ the PIVOT App can produce a continuous variable that can be evaluated independently, without the need to classify into distinct groups.

The reduction values from the PIVOT App were associated with knee abnormality. The odds ratio (OR) indicated that for every 1-millimeter increase observed by the PIVOT App, the odds of the knee being classified as abnormal increased by a factor of 5.87. This is important in the context of a health care practitioner assessing a knee and making a confident clinical diagnosis. For example, the probability of classifying a knee as abnormal/severely abnormal if it had 3 mm of tibial reduction was 94.8%.

A comprehensive meta-analysis of the pivot shift test compared findings with and without anesthesia.^5^ Without anesthesia, the sensitivity of the pivot shift test is low (24%, 95% CI, 21-27), with a high specificity (98% [95% CI, 96-99]).^5^ Under anesthesia, sensitivity increases considerably (74% [95% CI, 71-77]), and specificity remains high (99% [95% CI, 96-100]).^5^ The sensitivity of the PIVOT App (73.17%), which was based on the agreement with the examiner's categorically assigned grades, is comparable to the sensitivity under anesthesia reported in the literature. This result is promising, given that 40 (66.7%) participants were analyzed in a clinical setting rather than under anesthesia. However, the specificity (73.42%) was substantially lower with the PIVOT App than the sensitivity of the manual test reported in the literature.^5^ This suggests that more false positives are reported by the PIVOT App, whereas false negatives are more common in the clinical setting.^11,35^

Previously published findings from the PIVOT App showed that tibial translation in grade 2 classified knees (3.6 ± 1.2 mm) was significantly greater than in grade 1 knees (2.7 ± 0.6 mm; *P* < .05).^15^ Results from our study showed a lower mean reduction in both grade 2 (1.56 ± 1.15 mm) and grade 1 (0.84 ± 0.65 mm; *P* = .002) categorized knees. This could be a result of interexaminer variability across studies, or of the fact that most participants were analyzed while awake in the clinical setting. Although a statistically significant difference was observed in this study, as well as previously in the literature,^15^ it raises the question whether these values are clinically meaningful to a health care provider who would be performing this test on patients.

Although there was no statistically significant effect of muscle activation as measured on EMG, there were still some notable findings and interpretations to consider. Fifty percent of the trials included participants with EMG activity <2.99% and 2.27% of the MVC-normalized ST and RF muscle activity levels, respectively. Because multiple pivot shift test trials were conducted, ST and RF muscle activity were recorded only when the pivot shift was successfully elicited. As such, it seems that successful pivot shift testing can be performed by an experienced clinician with minimal protective guarding of the RF and ST muscles. Future research should assess ST and RF activity in trials in which an examiner experiences some guarding. This can lead to a better understanding of what muscle groups are primarily responsible for guarding during the pivot shift test, as well as determining thresholds for what constitutes high or low levels of guarding based on %MVC. Nevertheless, based on other research in ACL-D individuals, the RF and ST muscles appear to be involved in protective guarding of the knee joint.^2,4,12^

### Limitations

Some limitations were present in this study, including highly controlled testing conducted in the most ideal setting that could be created in a clinical environment. The “efficacy” design could lead to an overestimation of the PIVOT App's accuracy. Another threat to internal validity is that the same examiner assessed all knees in the clinical setting (ACL-injured and control). In contrast, the anesthesia group included analyses from 2 examiners. The low frequency of grade 3 knees in clinical practice is also a limitation, as the sample size (n = 2) markedly reduced statistical power, likely affecting the statistical significance of grade 3 knees relative to other grades. Although the PIVOT App is noninvasive and does not require procedures such as pin-fixed markers,^15^ skin and subcutaneous tissue create a barrier between bony landmarks and the surface markers. This can also affect the initial marker placement and the accuracy with which the bony landmarks beneath the marker and the skin are represented.

Translating the use of the PIVOT App into clinical practice presents feasibility limitations due to factors such as examination room setup with softbox lighting, the need for a second trained person to video-record the knee, and the need for repeated measurements to minimize patient guarding. On average, in the clinic, the test had to be conducted 3 times per knee. For participants analyzed under anesthesia, typically only 1 video was taken per leg due to time constraints. As a result, only patients with a complete data set from both knees were included. Data were excluded if 1 or both videos (2 knees) had markers leaving the frame, background artifacts were picked up that were not markers, or the video was incorrect for testing synchronization. Of the 58 trials available from the OR, only 20 were usable due to video imaging errors, a significant limitation. No trials were excluded based on the reduction number calculated by the PIVOT App. The PIVOT App may be useful for research settings to quantify the pivot shift test objectively. However, given the time required for setup and the multifaceted nature of clinical decision-making, the clinical utility is unclear.

## Conclusion

The PIVOT App is a noninvasive method for assessing ACL laxity, providing an objective, continuous assessment of the pivot shift test. The PIVOT App was able to successfully categorize knees as normal or abnormal based on the IKDC classification system, and the tibial reduction from grade 1 to 3 showed a consistent incremental increase. The PIVOT App addresses multiple limitations of the current manual reference standard.
